# Beef Steer Performance on Irrigated Monoculture Legume Pastures Compared with Grass- and Concentrate-Fed Steers

**DOI:** 10.3390/ani12081017

**Published:** 2022-04-14

**Authors:** Lance R. Pitcher, Jennifer W. MacAdam, Robert E. Ward, Kun-Jun Han, Thomas C. Griggs, Xin Dai

**Affiliations:** 1 Amalgamated Sugar Company, Boise, ID 83709, USA; lance.r.pitcher@aggiemail.usu.edu; 2 Department of Plants, Soils and Climate, Utah State University, Logan, UT 84322, USA; 3 Department of Nutrition, Dietetics and Food Sciences, Utah State University, Logan, UT 84322, USA; robert.ward@usu.edu; 4 School of Plant, Environmental and Soil Sciences, Louisiana State University, Baton Rouge, LA 70803, USA; khan@agcenter.lsu.edu; 5 Division of Plant and Soil Sciences, West Virginia University, Morgantown, WV 26506, USA; tcgriggs@gmail.com; 6 Utah Agricultural Experiment Station, Utah State University, Logan, UT 84322, USA; xin.dai@usu.edu

**Keywords:** birdsfoot trefoil, cicer milkvetch, condensed tannin, meadow bromegrass, non-fiber carbohydrate

## Abstract

**Simple Summary:**

Most U.S. beef calves are finished on grain-based rations, but a small proportion are finished on grass-dominated pastures. A number of temperate perennial legumes thrive when cultivated under irrigation in the U.S. Mountain West, including alfalfa, sainfoin, birdsfoot trefoil and cicer milkvetch. With the exception of alfalfa, these are non-bloating legumes, and all have fiber and non-fiber carbohydrate concentrations more similar to feedlot rations than to grass. This 3-year study was undertaken to compare the average daily gain of steer calves on monoculture legume pastures to the gain of calves on concentrate diets; in the final year, a grass pasture treatment was added. Gains of calves on birdsfoot trefoil pastures were two-thirds to three-quarters that of concentrate-fed calves; gains on cicer milkvetch pastures were about half that of concentrate-fed calves, and gains on grass pasture were about one-third that of concentrate-fed calves. The elevated gains of birdsfoot trefoil-fed calves compared with grass-fed calves were likely due to greater intake combined with the presence of a condensed tannin that precipitates excess plant protein in the rumen but does not interfere with post-ruminal digestion. Perennial legume pastures are a sustainable alternative to feedlot finishing for western U.S. beef producers.

**Abstract:**

Fall- or spring-born steers grazed monoculture irrigated birdsfoot trefoil (BFT; *Lotus corniculatus* L.) or cicer milkvetch (CMV; *Astragalus cicer* L.) pastures for approximately 12 weeks for 3 years and were compared with steers on concentrate diets. In the 3rd year, an irrigated meadow bromegrass (MBG; *Bromus biebersteinii* Roem. and Schult.) pasture treatment was added for further comparison. Steer average daily gain (ADG) was 1.31, 0.94, 0.83 and 0.69 kg d^−1^ on concentrate, ‘Norcen’ BFT, ‘Oberhaunstadter’ BFT, and ‘Monarch’ CMV diets, respectively; ADG on grass pastures was 0.43 kg d^−1^. The ADG on the concentrate diet was greater than ADG on legume or grass pastures, ADG was greater on BFT than CMV in every year (*p* < 0.03), and ADG on BFT was greater than ADG on grass (*p* < 0.03). The rate constant of gas production of an in vitro rumen fermentation demonstrated a slower rate of microbial digestion for CMV than for BFT. The elevated ADG on BFT pastures may be due to greater non-fiber carbohydrate (NFC) concentration and reduced neutral detergent fiber (NDF) concentration combined with condensed tannins that protect proteins in the rumen but do not impede protein digestion in the abomasum and intestines.

## 1. Introduction

Most ruminant production occurs on grasslands [1], and compared with grass-based production, legume-based production can provide advantages, including biologically fixed nitrogen and the increased digestibility and intake [2] that result in reduced methane emissions as a function of intake [3]. The presence of condensed tannins, which are secondary metabolites, makes temperate legumes non-bloating, decreases protein degradation in the rumen, reduces the nitrogen concentration of urine by partitioning more nitrogen into feces, and may function as an anthelmintic [4]. The elevated non-fiber carbohydrate concentration of perennial legumes grown in the Mountain West [5,6], where the growing season is characterized by long sunny days and cool nights, increases beef cattle nitrogen retention to levels normally seen only with grain supplementation [7].

A preliminary study of 300 kg calves [8] demonstrated average daily gain (ADG) on birdsfoot trefoil (BFT; *Lotus corniculatus* L.) pastures of 1.3 and 1.5 kg d^−1^ for the cultivars ‘Norcen’ and ‘Oberhaunstadter’, respectively and 1.2 kg d^−1^ for calves grazing ‘Monarch’ cicer milkvetch (CMV; *Astragalus cicer* L.). Birdsfoot trefoil is non-bloating because tannins precipitate soluble proteins in the rumen, whereas CMV is non-bloating because of the structure of its leaves [9]. Although BFT does not persist well in humid climates [10], it is productive and persistent when grown under irrigation in the Mountain West. The goal of the present study was to determine the season-long ADG of cattle grazing BFT or CMV pastures in comparison with cattle fed a concentrate-based diet in confinement. In the final year of the study, a grass treatment was also included. Our objectives were to document forage legume production and nutritive value, BFT tannin concentrations, in vitro digestibility, and ADG of fall- and spring-born steers, and compare the ADG and blood plasma fatty acid composition of concentrate- and pasture-fed cattle.

## 2. Materials and Methods

### 2.1. Pasture Establishment

Legume pastures were located at the Utah State University (USU) Caine Dairy Research Center in Wellsville, Utah (latitude 41°39′ N, longitude 111°54′ W; elevation 1386 m a.s.l.), concentrate feeding occurred at the USU Animal Science Farm, (latitude 41°40′ N, longitude 111°53′ W; 1370 m a.s.l.), and a grass pasture treatment was added in the last year of the study at the USU Intermountain Irrigated Pasture Project farm in Lewiston, Utah (latitude, 41.95° N; longitude, 111.87° W; elevation 1370 m a.s.l.). Legume pastures were designed as a randomized complete block with 6 replications. Each replication consisted of 3 contiguous pastures totaling 1.15 ha; pastures within each replication measured approximately 0.38 ha and were randomly assigned to 1 of 3 pasture treatments: Norcen or Oberhaunstadter (Ober) BFT or Monarch CMV. On 25–26 May 2010, BFT was broadcast seeded at 18 kg/ha pure live seed (PLS) and CMV at 30 kg/ha PLS. Pastures were irrigated biweekly using a K-Line pod sprinkler irrigation system (K-Line Irrigation/North America, St. Joseph, MI, USA); the study area was triangular, so irrigation lines served from 1 to 5 pastures. Five 0.36-ha grass pastures were broadcast seeded 14–15 August 2012 with meadow bromegrass (MBG) at 37 kg/ha PLS; these pastures were irrigated biweekly in 2013 using hand lines.

### 2.2. Grazing and Feeding Management

The number of groups of cattle assigned to each treatment decreased each year as steer BW increased. In 2011, 48 fall-born Angus steers with a mean weight of 302 ± 7 kg SEM were sorted by body weight (BW), and 12 cattle with similar total BW were randomly assigned to each of the three legume pasture treatments or the concentrate treatment. The treatment cattle were sorted by BW into four groups of three steers with similar total BW. Each pasture replication was divided into eight paddocks in 2011, and one paddock was grazed by a group of three cattle for 3.5 days; after 7 days, cattle were moved to a new replication of the same pasture treatment. In 2012, a similar procedure was followed with 36 fall-born Angus steers with a mean weight of 321 ± 4 kg SEM, with nine cattle assigned to each to the three legume pasture treatments or the concentrate treatment. Pasture replications were divided into six paddocks in 2012, and one paddock was grazed by a group of three cattle for 3.5 days; cattle were moved to a new replication of the same pasture treatment after 7 days. In 2013, 34 spring-born Angus steers with a mean body weight of 451 ± 5 kg SEM were divided among 5 treatment groups having similar body weight ranges. Of these cattle, 24 steers were sorted by BW and six steers with similar total BW were randomly assigned to one of the three legume pasture treatments or the concentrate treatment and divided into two groups of three steers with similar total BW. Pasture replications were divided into four paddocks, and each paddock was grazed by a group of three cattle for 3.5 days; cattle were moved to a new replication of the same pasture treatment after 7 days.

In 2013, the remaining 10 steers were assigned to the grass treatment and continuously grazed a single endophyte-free tall fescue pasture for the first 3 weeks of the grazing period because MBG pastures were newly established; cattle were moved to MBG pastures for the remaining 9 weeks of the study. Pairs of steers with similar total BW were randomly assigned to one of five MBG pasture replications [3]. Each replication was subdivided into 12 paddocks, and pairs of steers were moved to a fresh paddock every 3.5 days within the replication. Every occupied legume or grass paddock and feedlot pen was supplied at all times with fresh water and a trace mineralized salt block (Morton iOFIXT T-M; 970–985 g kg^−1^ NaCl, 3.5 g kg^−1^ Zn, 2.8 g kg^−1^ Mn, 1.75 g kg^−1^ Fe, 0.35–4.5 g kg^−1^ Cu, 0.07 g kg^−1^ I, and 0.07 g kg^−1^ Co).

In each year, each group of three steers in the concentrate treatment was randomly assigned to a separate pen at the USU Animal Science Farm. Cattle used in this study were not implanted with growth hormones or fed growth-stimulating feed additives. In 2011 and 2012, cattle were treated every 4 (2011) or 3 (2012) weeks with a liquid spray to reduce flies, and in 2013, cattle were given Permethrin^®^ ear tags (Y-Tex GardStar Plus, Y-Tex Corporation, Cody, WY, USA) at the beginning of the grazing season.

### 2.3. Data Collection

Cattle were shrunk overnight and weighed on 2 consecutive days at the beginning and end of the study each year, and again every 28 days in 2011 or every 21 days in 2012 and 2013 to determine ADG; the first period on pasture or concentrate diets each year was treated as an adjustment period and was not included in ADG calculations. Cattle grazed for 11 weeks in 2011, and for 12 weeks in 2012 and 2013. Blood samples for plasma fatty acid composition were taken at the beginning and end of dietary treatments. Blood was collected from the caudal vein of each animal in 10 mL BD Vacutainer^®^ tubes lined with 18 mg K_2_EDTA and immediately placed on ice. Blood samples were centrifuged at 1200× *g* for 10 min at 4 °C, and 0.5 mL aliquots of plasma were stored at −80 °C until analyzed.

Each week before cattle were moved to a new legume pasture replication or a new grass paddock, the next paddocks to be grazed in each treatment was sampled for forage dry matter (DM) and nutritive value. Forage DM was nondestructively estimated using a rising plate meter (RPM) calibrated for each pasture species. Samples for DM calibration were collected from each replication of each pasture each week by clipping the area under the RPM to a 1 cm height. To estimate pre- and post-grazing DM, paddocks were walked in a “lazy-W” pattern, and at least 30 RPM measurements were collected. Forage nutritive value samples were collected by walking a transect across ungrazed paddocks while clipping handfuls of grazable forage to approximately 7.5 cm every few steps. Rising plate meter calibration samples and forage quality samples were dried to constant weight at 60 °C. In 2012 and 2013, samples of seeded plant species to be used for tannin analysis were also collected along a transect across an ungrazed paddock of each replication. Tannin samples were frozen in the field between blocks of dry ice, then freeze-dried and ground to pass the 1 mm screen of a Wiley mill (Model 4, Thomas Scientific, Swedesboro, NJ, USA) before assay.

### 2.4. Forage Nutritive Value Analysis

Dried herbage biomass from each harvest date and treatment as well as forage nutritive value was determined via NIRS with a scanning monochromator (Model 6500, FOSS NIRSystems, Inc., Eden Prairie, MN, USA) and chemometrics software (WinISI ver. 4.5, Infrasoft International LLC, State College, PA, USA). Reflectance data were obtained at 2 nm increments between 1100 and 2498 nm. A mixed hay equation (12mh50-2.eqa, NIRS Forage and Feed Testing Consortium, Hillsboro, WI, USA) developed according to procedures of Shenk and Westerhaus [11] from a calibration set containing multiple species was used to predict sample composition. 

In vitro true DM digestibility (IVTDMD) of near-infrared reflectance spectroscopy (NIRS) calibration samples was determined by incubating samples in buffered rumen fluid for 48 h followed by refluxing of indigestible residues in neutral detergent solution [12,13]. Digestible neutral detergent fiber (NDF) (dNDF, as a proportion of DM) and neutral detergent fiber digestibility (NDFD, as a proportion of NDF) were calculated from amylase-treated NDF (aNDF) and IVTDMD concentrations [13]. For 48-hr incubation times, values of IVTDMD are approximately 12 percentage units greater [14] than those of in vitro apparent digestibility for the same samples analyzed by the traditional 2-stage procedure of Tilley and Terry [15]. Determination of acid detergent fiber (ADF), crude protein (CP), NDF, acid detergent lignin (ADL) and ash of calibration samples was made according to AOAC International [16] methods 973.18, 984.13, 2002.04, 973.18 and 942.05, respectively, and crude fat (CF) by AOAC [17] method 920.39. Rumen-undegradable protein (RUP) concentration as a proportion of CP was determined according to Hoffman et al. [18], and non-fiber carbohydrate (NFC) concentration was calculated similarly to NRC [19] as 100-((NDF-2.0) + CP + 2.5 + ash), which assumes concentrations of 2.0 and 2.5% for neutral detergent insoluble CP [13] and fat, respectively, in forage samples. The distribution and boundaries of BFT, CMV and MBG sample spectra were well-represented by the population structure of spectra in the calibration set, so no additional wet chemistry was required.

### 2.5. Forage Fermentation

Ground, freeze-dried forage legume samples (500 mg) were weighed into 250 mL incubation bottles. Buffered mineral solution [12] was prepared, and 16 mL of the solution was distributed to the incubation bottles in a water bath at 39 °C. Rumen fluid was collected after the morning feeding from a ruminally fistulated Holstein heifer fed alfalfa and grass hay twice daily, at 07:00 and 18:00 h. Rumen fluid was obtained with a 300 mL plastic beaker and transferred into two pre-warmed air-tight thermos containers. Collected rumen fluid was immediately moved into a laboratory and filtered through four layers of cheesecloth and flushed with carbon dioxide (CO_2_). Rumen fluid was combined with the remainder of the prepared buffered mineral solution, which was maintained in a water bath at 39 °C. All handling was under continuous blanket flushing with CO_2_. About 24 mL of buffered rumen fluid was dispensed into 250 mL incubation bottles containing forage samples. After assembling each bottle to a module (Ankom Technology, Macedon, NY, USA) capable of communicating with a computer using radio frequency transmission, bottles were gently shaken. The whole incubation was processed at 39 °C, and gas readings for each bottle were recorded at 30 min intervals.

Rate and extent of gas production were determined for each forage sample by fitting the gas production data to the dual pool logistic equation [20]:

V = V_F1_ (1 + exp (2 + 4 µ_m1_/V_F1_ × (λ_1_ − t)))^−1^ + V_F2_ (1 + exp (2 + 4 µ_m2_/V_F2_ × (λ_2_ − t)))^−1^ (1)

where V is the amount of gas production at time t, and V_F1_ and V_F2_ are the final gas production volumes corresponding to complete substrate digestion for a rapidly fermenting pool and a slowly fermenting pool, respectively. µ_m1_ and µ_m2_ are the points of inflection of the gas curve for the two pools, respectively; λ_1_ and λ_2_ are the lag times of the two pools.

#### Tannin Analysis

Forage samples were analyzed for condensed tannins by the hydrochloric acid (HCl)- butanol-acetone-iron method of Grabber et al. [21]. The assay solution contained 0.15% *w/v* ammonium iron (III) sulfate dodecahydrate, 3.3% *v/v* water, 5% *v/v* concentrated HCl, 41.7% *v/v* butyl alcohol, and 50% *v/v* acetone. Briefly, triplicate 0.030 g DM of ground plant tissue was suspended in 15 mL of tannin assay solution and heated for 2.5 h in a 70 °C water bath. Samples were mixed periodically during heating. Standard, blank solutions and check samples were included in each run. After tubes cooled, they were centrifuged at 5000× *g* for 10 min and the absorbance of the supernatant was determined at 554 nm.

Tannin used as the standard for the spectrophotometric assay was isolated as described by Hagerman [22] from the Oberhaunstadter cv. of L. corniculatus. Briefly, a suspension of 5% *w/v* finely ground plant material in 1% *v/v* acetic acid, 24% *v/v* water and 75% *v/v* acetone was sonicated for 30 min with periodic mixing. Mixtures were centrifuged for 10 min at 3000 × *g* and the supernatant filtered through a coarse fritted disk; plant material was extracted a total of 3 times and supernatants combined. The supernatant was mixed with an equal volume of ethyl ether, and the aqueous layer was retained; the supernatant was extracted a total of 3 times with equal volumes of ethyl ether. The acetone and ethyl ether remaining in the aqueous solution were removed by rotary evaporation. The aqueous solution was mixed with Sephadex LH 20 resin equilibrated in a 4:1 *v/v* ethyl alcohol:water solution, rinsed with 95% ethyl alcohol, and extracted with a 3:1 *v/v* acetone:water solution. The acetone was removed by rotary evaporation, and the aqueous solution was frozen and freeze-dried.

### 2.6. Blood Fatty Acid Analysis

Blood fatty acids were analyzed according to the method of O’Fallon et al. [23] with slight modifications. Briefly, 100 µL of plasma or forage extract was added to a screw cap tube along with 100 µL of internal standard (0.5 mg/mL C19:1 triglyceride in chloroform), 700 µL of 10 M potassium hydroxide (KOH) and 5.3 mL of methanol. Samples were incubated for 1.5 h at 55 °C in a shaking water bath, after which tubes were cooled on ice before 570 µL of 12 M sulfuric acid (H_2_SO_4_) was added. Tubes were inverted several times to mix thoroughly and then placed back in the shaking water bath at 55 °C for 1.5 h. Tubes were cooled on ice, 3 mL of hexane was added and samples were vortexed for 30 s. Samples were centrifuged for 5 min at 1000× *g* to separate phases, and 1 mL of the hexane layer was pipetted into a sample vial for gas chromatographic analysis.

Fatty acid methyl esters were analyzed using a Shimadzu GC2010 gas chromatograph (Shimadzu Scientific Instruments, Columbia, MD, USA) equipped with an HP-88 capillary column (100 m × 0.25 mm i.d. × 0.2 µm film thickness; Agilent Technologies, Santa Clara, CA, USA) and a flame ionization detector. The injector was maintained at 250 °C and a head pressure of 206.7 kPa, and 1 µL of sample was injected at a split ratio of 10:1. Hydrogen was used as the carrier gas at a linear flow rate of 31.1 mL/min. The oven program was as follows: initial temperature 35 °C for 2 min, ramp at 40 °C/min to 175 °C, hold 4 min, ramp at 3.5 °C/min to 250 °C and hold for 25 min. The flame ionization detector was operated at 250 °C, with air and hydrogen supplied at 400 and 39 mL/min, respectively. A standard mixture of fatty acids (GLC-663; NuChek Prep, Elysian, MN, USA) was used for analyte identification by retention time and for the generation of response factors. Fatty acid data are expressed as a percentage of all fatty acids detected (g/100 g fat).

### 2.7. Statistical Design and Analysis

Experimental units comprised groups of three cattle and their assigned paddocks on legume pastures, groups of three cattle and their assigned pens for the concentrate treatment, and groups of two cattle and their assigned paddocks on grass pastures. Forage pre-grazing and post-grazing dry matter, forage quality and tannin concentrations were analyzed using mixed models with year and species classified as fixed effects and using pre-grazing dry matter as a covariate for post-grazing dry matter, with week (forage quality) or grazing unit as random blocks. 

A mixed model was used to calculate ADG in which year, treatment, days in study and initial weight were fixed factors and group (replication) was a random factor. Although year and treatment were classified factors, days in study and initial weight were continuous factors; ADG was therefore the slope of days in study. Two-way and three-way interactions between year and treatment with days in study and with initial weight were tested. The ante(1) covariance structure was applied to model residuals to account for the correlations among repeated measurements.

Steer blood fatty acid components were analyzed using a mixed model with year, treatment and date (start, end) as classified fixed effects. Year and treatment were tested at the group level and date was tested at the animal level. The residual covariance structure was compound symmetry to account for the repeated measures on each animal. Each of the 7 blood fatty acid components was analyzed separately. All analyses were conducted using PROC GLIMMIX of SAS/STAT 13.2 (SAS Institute, Cary, NC, USA). We used the ESTIMATE statement in the procedure to compare the slopes for ADG and to compare the Least Squares Means (LSMEANS) of interest for blood fatty acid components.

## 3. Results

**Figure 1:**
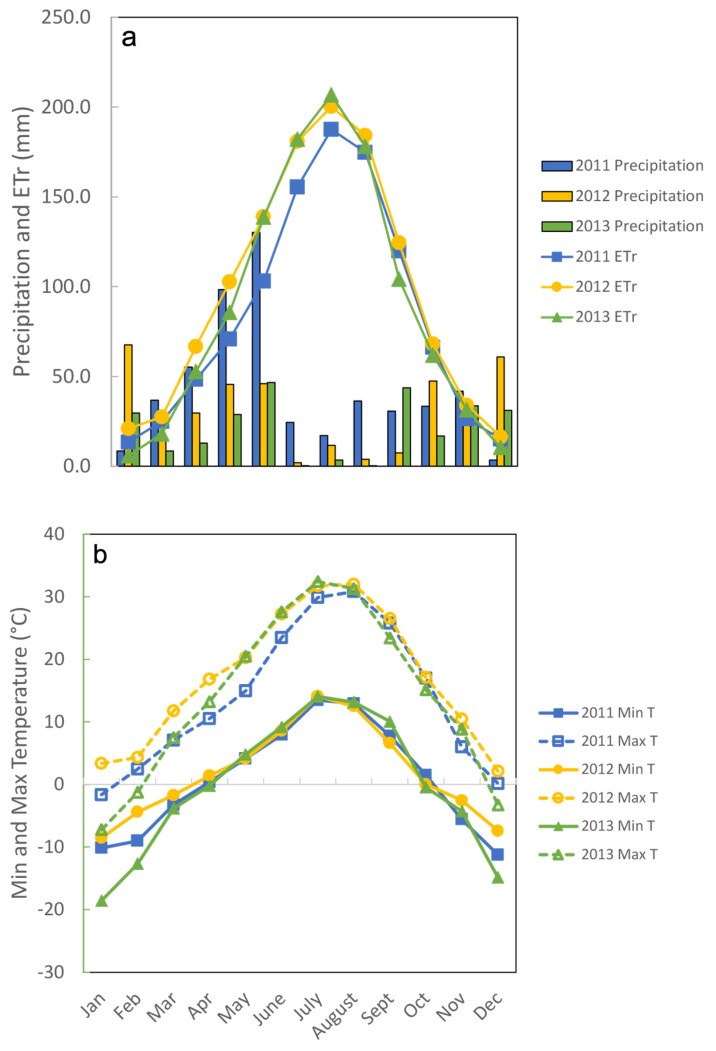
(**a**). Precipitation (columns) and evapotranspiration (lines) during the 3 years of the study. (**b**). Minimum (solid lines) and maximum (dashed lines) temperatures during the 3 years of the study.

Forage utilization was maintained between 50 and 60% of pasture DM (Table 1) by reducing stocking density from four (2011) to three (2012) to two (2013) groups of cattle on legume treatments as cattle weights increased. Pre-grazing DM was greater for CMV than for Norcen BFT in 2013 (*p* = 0.0265). For post-grazing DM and forage DM disappearance, the main effect of year was not significant: post grazing DM was greater for Ober BFT than for CMV (*p* = 0.0150) and DM disappearance was greater for CMV than for Ober BFT (*p* = 0.0150). In all 3 years, all paddocks were grazed twice between May and August, and the dry matter remaining after grazing was approximately 2000 kg/ha, allowing cattle to be relatively selective. Climate data illustrate the difference between precipitation and evapotranspiration during the May to August grazing periods each year (Figure 1a) and the approximately 20 °C decrease in nighttime temperatures during the hottest part of the summer (Figure 1b).

Comparing the forage nutritive value of the two legumes (Table 2), CMV and BFT had similar CP, aNDF and ADF, whereas CMV had greater IVTDMD, dNDF, and NDFD (*p* < 0.01), than BFT, suggesting that the intake of cattle grazing CMV might exceed that of cattle grazing BFT, which was the case in 2011 and 2013 (Table 1). Comparing MBG with the legumes in 2013, the CP and NFC were less and the aNDF and ADF were greater for the grass than for the legumes. The aNDF and NFC values of the three legumes were similar to those of the concentrate diet, which contained 15% CP, 31% NDF, and 43% NFC [5].

In 2012 and 2013, pasture species were assayed for condensed tannin concentration (Table 3). In both years, tannin concentrations of Norcen and Oberhaunstadter BFT ranged from 11 to 14 g/kg DM and were not significantly different. Cicer milkvetch [24] and other *Bromus* species [25] are not reported to contain condensed tannins but do react minimally with the tannin assay solution. Therefore, tannin concentrations were greater for BFT than for CMV and MBG (*p* < 0.0001).

For ADG (Table 4), F-tests showed a significant interaction effect of days in study with treatment (*p* < 0.0001). Year-by-treatment interaction was also highly significant (*p* = 0.02). Other interactions were not significant at α = 0.05. ADG for each treatment was estimated by obtaining separate slopes of days in study under each treatment. Initial weight significantly affected animal weight measurements during the experiment. The estimated coefficient was 0.92 ± 0.02 (*p* < 0.0001) which means 1 kg initial weight change would result in an average 0.92 kg weight change during the experiment period in the same direction.

**Figure 2:**
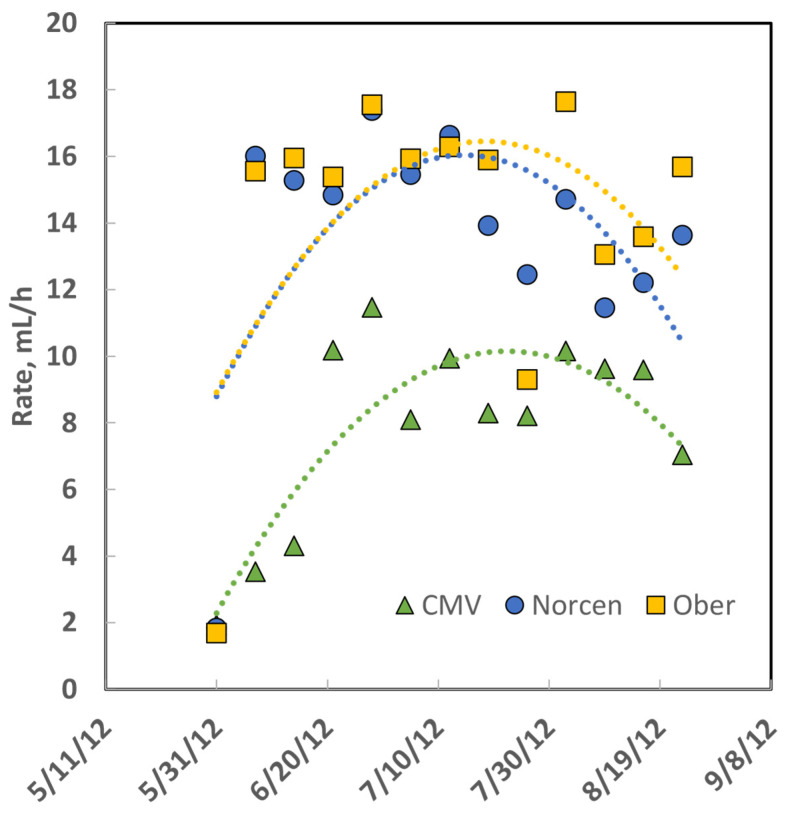
Rate constant data for gas production during in vitro forage fermentation of samples collected from rotationally stocked pre-grazing pastures in 2012; dates are formatted as month/day/year. Data were fitted with second-order polynomial equations, illustrating that the magnitude of fermentation rate constants of the two birdsfoot trefoil (BFT) cultivars were similar to each other and greater than cicer milkvetch (CMV).

The ADG of steers grazing Norcen BFT pastures was greater than that of steers grazing CMV pastures in all 3 years of the study (Table 4), whereas the gain of steers on Ober BFT was more variable: gain on Ober was greater than for steers on Norcen BFT in 2011, no different in 2012, and less for Ober than Norcen steers in 2013. The ADG of cattle on the concentrate diet was always greater than the ADG of cattle on legume and grass pastures. The rate constant of gas production [26] from in vitro rumen fermentation of the three legumes (Figure 2) demonstrates that CMV is digested more slowly than BFT even though it had greater predicted dNDF and IVTDMD.

Blood plasma fatty acid variables did not differ among cattle at the beginning of the study in any year (Table 5, Table 6 and Table 7). In 2011 and 2013, steers were fed a mixture of alfalfa hay and corn silage before being placed in the grazing study. In 2012, no corn silage was fed and this was reflected in the elevated omega-3 and reduced omega-6 fatty acid concentrations in blood plasma at the start of grazing that year. At the end of each grazing season, all steers on pasture diets had smaller (more favorable) omega-6 to omega-3 ratios (*p* < 0.0001) in their blood plasma, between 1.5 and 2.8, whereas concentrate-fed cattle had greater omega-6 levels and reduced omega-3 levels after 3 months on concentrates. The omega-6 to omega-3 ratios of concentrate-fed cattle at the end of the study period ranged from 3.2 to 11.3, increasing as initial cattle BW increased. The concentrations of omega-3 fatty acids were greater (*p* < 0.0001) and the concentrations of omega-6 fatty acids were less (*p* < 0.01) for pasture than for concentrate diets at the end of each study period in all three years. The concentrations of TVA were greater (*p* < 0.0001) for pasture than concentrate diets in all three years except one instance in for 2011, when the *p*-value of the difference between CMV and the concentrate diet was 0.0156.

## 4. Discussion

### 4.1. Forage Production and Quality

Although pasture utilization was similar from year to year, legume pre-grazing DM increased 23% from year 1 to year 2, and 9% from year 2 to year 3, which is typical for the development of perennial forage stands in the years following planting. In a Utah BFT variety trial [27] forage production increased for the first 2 years after planting. In a Missouri study [10] the forage production of BFT pastures was 3304 kg/ha in the year after planting and 4266 kg/ha in the following year, a 29% increase.

The greater digestibility and intake of legumes cultivated under irrigation in the Mountain West can result in greater ruminant productivity on forage legumes compared with grasses [28,29,30]. The season-long NDF concentration of pre-grazing Norcen BFT pastures in this study ranged from 25.8 to 30.7%, whereas the NDF of Norcen in a spring beef grazing study in Missouri ranged from 50.3 to 58.4% [10]. In 2013, the NDF of Cache meadow bromegrass was 50.4%; in the Missouri study, spring tall fescue NDF ranged from 64.9 to 74.7%. By comparison, the NDF of the concentrate diet fed to control steers was 31.0 and the NFC was 42.7%. The mean NFC of the three legumes in the 3 years of this study was 39%, whereas that of meadow bromegrass was 19% in 2013. Although both legumes and grasses accumulate less NDF when grown under irrigation in the Mountain West than in the more humid midwestern US, the elevated NFC concentrations of perennial legumes grown in both pastures and for hay [7] is comparable to that of beet pulp or corn silage [31]. This is likely the result of the long intensively sunny days (maximal photosynthesis) and cool nights (minimal aerobic respiration) typical of the Mountain West climate.

Birdsfoot trefoil tannins are present in a relatively low concentration (1–3%; [32]). Although they bind excess plant proteins in the rumen, proteins associated with BFT tannins become available for digestion in the abomasum [33]. A review by Waghorn [4] concluded that the condensed tannins synthesized by BFT increased utilization of proteins in the abomasum, resulting in better feed efficiency and increased absorption of amino acid compared with other tannin-containing forages. In studies carried out in other climates, the BFT cultivar Oberhaunstadter was reported to accumulate more tannin than the cultivar Norcen [34,35]. In the present study, the tannin concentrations of the two cultivars did not differ, but the elevated NFC concentrations of BFT may serve to dilute tannin concentrations as a proportion of herbage DM. Greater meat and milk production have been reported for ruminants grazing BFT compared with alfalfa [36,37,38]. The elevated level of readily digested carbohydrates combined with the protection of soluble protein from digestion in the rumen appeared in this study to result in greater ADG for steers grazing BFT than either CMV or a high-quality grass. Cicer milkvetch has been found to contain a water-soluble arabinogalactan protein that prevents cellulolytic bacteria from adhering to cellulose [39,40], which likely reduced the maximum rate of in vitro fermentation of CMV relative to BFT in this and other studies [41].

### 4.2. Beef Steer Production

Over the 3 years of this study, cattle on Ober and Norcen BFT gained two-thirds and three-quarters, respectively, of the amount gained by concentrate-fed cattle. The ADG of CMV-fed cattle was half that of cattle fed a concentrate diet, whereas grass-fed cattle gained about one-third that of concentrate-fed cattle. Wen et al. [10] reported spring gains of 1.26 to 1.53 kg/d on pure stands of Norcen BFT in Missouri and MacAdam et al. [8] reported gains of 1.30 kg/d on pure stands of Norcen BFT for 61 to 77 days of grazing. Gains reported by other studies of cool-season perennial grass pastures have been as high as 0.84 kg/d for tall fescue [42] and smooth bromegrass (*Bromus inermis* Leyss.; [37]). On grass-legume mixtures, gains of 0.87 kg/d for tall fescue-BFT [10], 0.97 kg/d for smooth brome-BFT pastures [37], and 1.81 kg/d for early season irrigated white clover-orchardgrass (*Dactylis glomerata* L.) pastures [43] have been reported. In a typical feedlot setting, ADG for cattle reportedly range from 0.79 to 2.43 kg/d for 90 to 120 days [44]; our concentrate treatment gains increased with initial steer weight, ranging from 1.14 to 1.57 kg/d with no growth-promoting implants or feed additives. In 2013, cattle from the concentrate, Norcen BFT and MBG treatments were slaughtered in mid-September and used in a carcass evaluation and sensory panel study [5]. The tenderness and juiciness of ribeye steaks from legume-finished cattle were comparable to steaks from concentrate-finished cattle, and overall, legume- and concentrate-finished steaks were comparably well-liked. For most characteristics, legume- and concentrate-finished steaks were preferred to steaks from grass-finished cattle. Data from 2013 were also used to calculate the number of cattle needed to produce 1 billion kg of red meat. It was determined that the elevated dressing percentage of BFT-finished cattle meant only 5% more legume-finished than concentrate-finished cattle would be required to yield 1 billion kg of red meat, whereas 25% more grass-fed cattle would be required to yield the same amount of red meat [45].

### 4.3. Blood Plasma Fatty Acids

In studies of other tannin-containing legumes, the fatty acid composition of blood plasma predicted trends in the fatty acid composition of intramuscular fat [46]. At the end of the grazing season, blood plasma TVA was greater in pasture-fed than concentrate-fed steers, regardless of pasture treatment. TVA is produced in the rumen through biohydrogenation of mono- and polyunsaturated substrates, including both linoleic and linolenic acids; linolenic acid is more efficiently converted to TVA than linoleic acid [47]. At the end of the grazing period, cattle on the pasture treatments had greater blood plasma TVA concentrations than cattle on concentrate diets. In 2013, cattle from the concentrate, BFT and grass pasture treatments remained on treatments until they were slaughtered in mid-September and subjected to carcass evaluation. The meat from these cattle was evaluated by consumer sensory panels and quality characteristics were determined. TVA is a precursor for conjugated linoleic acid (CLA), but differences in TVA did not result in differences in the CLA of intramuscular fat of ribeye steaks [5].

## 5. Conclusions

This study illustrates the relatively high forage quality of perennial legume pastures cultivated under irrigation in the Mountain West, with NDF and NFC comparable to that of a concentrate diet. Steer ADG on the two BFT cultivars averaged 0.89 kg/d, whereas that of steers on concentrate diets averaged 1.31 kg/d. Steers grazing the other legume, CMV, gained 0.69 kg/g, and in the last year of the study, grass-fed steers gained 0.43 kg/d. The in vitro fermentation rate of CMV was less than that of both BFT cultivars, possibly due to the accumulation of an arabinogalactan protein in CMV that interferes with the digestion of cellulose. The blood plasma concentration of omega-6 fatty acids was reduced and the concentration of omega-3 fatty acids was increased for all pasture-fed steers compared with concentrate-fed steers, resulting in a more favorable omega-6 to omega 3 fatty acid ratio. The high quality of birdsfoot trefoil pastures significantly increased gain of cattle compared with cattle on both cicer milkvetch and meadow bromegrass pastures.

## Figures and Tables

**Table 1:** LSMEANS of season-long forage dry matter (DM) measured before (pre) and after (post) grazing, forage disappearance, calculated as the difference between pre- and post-grazing DM, and pasture utilization.

	Pre	Post	Forage Disappearance	Utilization
Diet	kg/ha			%
2011				
Ober BFT	4540	2270 a	2322 b	51
Norcen BFT	4075	2046 ab	2080 c	51
CMV	4488	1890 b	2610 a	59
SEM	285	105	105	
2012				
Ober BFT	5476	2535	2847	52
Norcen BFT	5445	2456	2895	53
CMV	5169	2165	2910	56
SEM	272	105	105	
2013				
Ober BFT	5752 ab	2782 ab	3022 b	53
Norcen BFT	5517 b	2682 b	2887 b	52
CMV	6347 a	2898 a	3501 a	55
MBG	4075 c	1671 c	2404 c	57
SEM	285	105	105	

LSMEANS, least squares means; BFT, birdsfoot trefoil; CMV, cicer milkvetch; MBG, meadow bromegrass, used for the last 6 wk of 2013. Within each year, values in columns with different letters are significantly different at *p* < 0.05.

**Table 2:** LSMEANS of season-long forage nutritive value characteristics.

	CP	aNDF	ADF	IVTDMD	dNDF	Ash	Fat	ADL	NFC	NDFD	RUP
Diet	% DM									% NDF	% CP
2011											
Ober BFT	23.8 a	31.5	28.7	81.8 b	14.0 b	8.6 b	1.8	4.3 a	37.0	45.4 b	17.1
Norcen BFT	23.2 ab	30.2	27.8	82.6 b	13.6 b	8.6 b	1.9	4.2 a	37.9	45.5 b	17.1
CMV	21.3 b	31.9	28.6	87.9 a	20.5 a	9.5 a	1.8	3.9 b	37.2	65.5 a	16.9
SEM	1.07	1.27	1.02	1.12	0.68	0.33	0.07	0.16	0.89	2.31	0.77
2012											
Ober BFT	26.3 ab	24.7	22.3	86.7 b	11.3 b	7.3 b	2.0 b	3.8 a	41.5 a	45.8 b	17.6
Norcen BFT	26.0 b	25.8	22.6	85.8 b	11.9 b	7.0 b	2.1 ab	3.8 a	41.3 a	47.5 b	17.6
CMV	27.8 a	24.8	21.9	93.3 a	18.1 a	8.1 a	2.2 a	3.4 b	39.2 b	73.4 a	16.6
SEM	0.85	1.00	0.80	0.90	0.57	0.27	0.06	0.12	0.74	1.89	0.65
2013											
Ober BFT	23.8 b	29.0 b	26.0 b	83.4 b	12.8 d	6.1 c	2.0 b	3.9 a	41.0 a	44.2 c	18.8 b
Norcen BFT	23.7 b	30.7 b	26.2 b	83.8 b	14.9 c	6.5 c	2.1 b	3.9 a	39.0 b	48.7 b	18.8 b
CMV	27.0 a	27.3 b	23.7 c	91.4 a	19.0 b	8.2 b	2.0 b	3.7 a	37.5 b	70.7 a	16.8 b
MBG	18.2 c	50.4 a	32.2 a	85.2 b	34.9 a	10.8 a	3.4 a	3.3 b	19.3 c	69.5 a	38.9 a
SEM	1.03	1.21	0.97	1.08	0.65	0.32	0.07	0.15	0.86	2.22	0.74

LSMEANS, least squares means; CP, crude protein; aNDF, neutral detergent fiber assayed with the addition of heat-stable α-amylase; ADF, acid detergent fiber; IVTDMD, in vitro true dry matter digestibility; dNDF, digestible NDF; ADL, acid detergent lignin; NFC, non-fiber carbohydrate; NDFD, NDF digestibility; RUP, rumen undegradable protein; BFT, birdsfoot trefoil; CMV, cicer milkvetch; MBG, meadow bromegrass, used for the last 6 wk of 2013. Within each year, values in columns with different letters are significantly different at *p* < 0.05.

**Table 3:** LSMEANS of season-long condensed tannin (CT) concentrations, measured in 2012 and 2013.

	2012		2013	
	CT	SEM	CT	SEM
Diet	g/kg DM			
Ober BFT	12.5 a	0.74	13.6 a	0.84
Norcen BFT	10.6 a	0.63	12.9 a	0.79
CMV	2.4 b	0.14	1.7 c	0.11
MBG			2.5 b	0.28

LSMEANS, least squares means; DM, dry matter; BFT, birdsfoot trefoil; CMV, cicer milkvetch MBG, meadow bromegrass. Values in columns with different letters are significantly different at *p* < 0.05.

**Table 4:** LSMEANS of season-long average daily gain (ADG).

	Average Daily Gain				
	2011	2012	2013	3-Year Mean	% of 3-Year Mean
Diet	kg/d				Concentrate ADG
Concentrate	1.14 a	1.21 a	1.57 a	1.31	
Ober BFT	0.95 b	0.88 bc	0.65 c	0.83	66
Norcen BFT	0.80 c	0.98 b	1.03 b	0.94	72
CMV	0.64 d	0.81 c	0.63 c	0.69	53
MBG			0.43 c		35
SEM	0.06	0.07	0.16		

LSMEANS, least squares means; BFT, birdsfoot trefoil; CMV, cicer milkvetch; MBG, meadow bromegrass. Values in columns with different letters are significantly different at *p* < 0.10.

**Table 5:** LSMEANS of 2011 cattle blood plasma fatty acid composition at the beginning (23 June) and end (9 September) of a 78-day feeding period on a concentrate diet or on perennial legume pastures.

		Concentrate	Ober BFT	Norcen BFT	CMV
Fatty Acid Component	Date	g/100 g Fat			
Saturated fatty acids	Start	34.3	35.0	34.3	35.1
	End	37.8 a	34.3 b	35.4 b	34.9 b
	End SEM	0.92	0.84	0.83	0.82
trans-Vaccenic acid (TVA)	Start	0.77	0.97	0.89	0.91
	End	0.37 c	0.72 b	1.09 a	0.60 b
	End SEM	0.052	0.099	0.144	0.079
Monounsaturated fatty acids	Start	13.8	14.8	14.0	14.6
	End	8.6 c	9.1 c	10.7 b	12.2 a
	End SEM	0.34	0.36	0.40	0.46
Polyunsaturated fatty acids	Start	45.5	43.2	45.5	43.9
	End	47.9	50.6	47.3	47.7
	End SEM	1.15	1.22	1.09	1.10
Total omega-6 fatty acids	Start	34.4	32.0	34.2	32.5
	End	37.9 a	33.2 b	31.5 b	30.6 b
	End SEM	1.25	1.09	1.00	0.97
Total omega-3 fatty acids	Start	10.7	10.9	11.0	11.1
	End	9.7 b	17.2 a	15.7 a	16.8 a
	End SEM	0.35	0.63	0.55	0.59
Omega-6 to omega-3 ratio	Start	3.2	3.0	3.1	2.9
	End	3.9 a	1.9 b	2.0 b	1.8 b
	End SEM	0.21	0.10	0.10	0.09

LSMEANS, least squares means; BFT, birdsfoot trefoil; CMV, cicer milkvetch. Values in rows with different letters are significantly different at *p* < 0.05.

**Table 6:** LSMEANS of 2012 cattle blood plasma fatty acid composition at the beginning (31 May) and end (23 August) of an 84-d feeding period on a concentrate diet or on perennial legume pastures.

		Concentrate	Ober BFT	Norcen BFT	CMV
Fatty Acid Component	Date	g/100 g Fat			
Saturated fatty acids	Start	35.2	36.2	35.4	35.3
	End	34.2	33.4	33.3	33.7
	End SEM	0.54	0.50	0.50	0.53
trans-Vaccenic acid (TVA)	Start	0.93	1.05	1.00	0.99
	End	0.56 b	1.27 a	1.24 a	1.24 a
	End SEM	0.042	0.090	0.087	0.093
Monounsaturated fatty acids	Start	12.4	12.1	12.5	12.5
	End	11.7 a	9.9 b	9.8 b	11.0 ab
	End SEM	0.60	0.48	0.47	0.56
Polyunsaturated fatty acids	Start	44.2	43.9	44.4	44.3
	End	48.9	50.4	50.3	48.6
	End SEM	1.05	1.03	1.02	1.03
Total omega-6 fatty acids	Start	23.8	24.3	24.5	24.8
	End	43.1 a	32.3 b	31.3 b	30.5 b
	End SEM	1.28	0.91	0.89	0.90
Total omega-3 fatty acids	Start	20.0	19.1	19.6	19.1
	End	5.6 b	18.0 a	18.8 a	17.8 a
	End SEM	0.13	0.41	0.43	0.43
Omega-6 to omega-3 ratio	Start	1.2	1.3	1.3	1.3
	End	7.7 a	1.8 b	1.7 b	1.7 b
	End SEM	0.32	0.07	0.07	0.07

LSMEANS, least squares means; BFT, birdsfoot trefoil; CMV, cicer milkvetch. Values in rows with different letters are significantly different at *p* < 0.05.

**Table 7:** LSMEANS of 2013 cattle blood plasma fatty acid composition at the beginning (31 May) and end (23 August) of an 84-day feeding period on a concentrate diet or on perennial legume and grass pastures.

		Concentrate	Ober BFT	Norcen BFT	CMV	MBG
Fatty Acid Component	Date	g/100 g Fat				
Saturated fatty acids	Start	33.9	35.5	33.9	34.7	34.6
	End	31.8	31.9	31.2	33.2	32.4
	End SEM	0.62	0.69	0.61	0.65	0.49
trans-Vaccenic acid (TVA)	Start	0.56	0.61	0.61	0.56	0.59
	End	0.13 c	1.16 b	1.04 b	0.85 b	2.20 a
	End SEM	0.017	0.166	0.136	0.112	0.234
Monounsaturated fatty acids	Start	21.0	19.5	18.4	20.9	20.7
	End	14.9 c	16.8 bc	15.8 c	18.9 ab	20.0 a
	End SEM	0.77	0.95	0.82	0.98	0.80
Polyunsaturated fatty acids	Start	40.1	39.6	41.9	39.4	39.5
	End	49.2 a	45.7 ab	47.2 a	42.1 b	38.0 c
	End SEM	1.50	1.53	1.44	1.28	0.90
Total omega-6 fatty acids	Start	29.6	29.0	30.8	29.4	29.3
	End	45.1 a	29.9 b	29.9 b	27.2 c	22.6 d
	End SEM	1.37	1.00	0.91	0.83	0.53
Total omega-3 fatty acids	Start	10.3	10.4	11.0	9.8	10.0
	End	4.0 c	15.6 ab	17.1 a	14.7 b	14.9 b
	End SEM	0.16	0.69	0.70	0.60	0.47
Omega-6 to omega-3 ratio	Start	2.9	2.8	2.8	3.0	2.9
	End	11.3 a	1.9 b	1.7 b	1.0 b	1.5 c
	End SEM	0.38	0.07	0.06	0.06	0.04

LSMEANS, least squares means; BFT, birdsfoot trefoil; CMV, cicer milkvetch; MBG, meadow bromegrass, used for the last 9 wk of 2013. Values in rows with different letters are significantly different at *p* < 0.05.

## Data Availability

The data that are summarized in this study are available on request from the corresponding author.
